# Efficacy and Safety of Endoscopic Intralesional Triamcinolone Injection for Benign Esophageal Strictures

**DOI:** 10.1155/2018/7619298

**Published:** 2018-08-06

**Authors:** Ya-Wu Zhang, Feng-Xian Wei, Xue-Ping Qi, Zhao Liu, Xiao-Dong Xu, You-Cheng Zhang

**Affiliations:** ^1^Department of General Surgery, Lanzhou University Second Hospital, Lanzhou 730000, China; ^2^Hepato-Biliary-Pancreatic Institute, Lanzhou University Second Hospital, Lanzhou 730000, China; ^3^Lanzhou University Second Clinical Medical College, Lanzhou University, Lanzhou 730000, China; ^4^The First Clinical Medical College of Lanzhou University, Lanzhou 730000, China

## Abstract

**Objectives:**

To evaluate the efficacy and safety of endoscopic intralesional triamcinolone injection (ITI) for benign esophageal strictures combined with endoscopic dilation (ED).

**Methods:**

Online databases including MEDLINE, EMBASE, the Cochrane Library, and Web of Science were comprehensively searched for prospective randomized control trials (RCTs) between 1966 and March 2018. A meta-analysis was conducted according to the methods recommended by the Cochrane Collaboration.

**Results:**

Six RCTs consisting of 176 patients were selected. Meta-analysis results showed that additional ITI had a significant advantage in terms of stricture rate and required ED sessions. Surgery-related and non-surgery-related strictures showed similar results. Additional ITI was not associated with significantly increased risk of complications.

**Conclusions:**

Our meta-analysis showed that additional ITI therapy was supposed to be effective and safe for benign esophageal strictures as it reduced the stricture rate and required ED sessions. However, more RCTs are necessary to support these findings.

## 1. Introduction

Surgical anastomosis, radiation therapy, Schatzki's rings, esophageal webs, corrosive injury, peptic injury, photodynamic therapy [1, 2], and endoscopic surgery can always induce benign esophageal strictures [3–5]. These injuries can induce edema, and finally lead to stricture formation through stimulating the proliferation of fibrotic tissue and/or accumulation of collagen [6]. Aside from resolving the severity of the stricture, the intended therapy also focused on the improvement of quality of life and avoidance of related complications, as well as the prevention of recurrences. Currently, endoscopic dilation (ED) is the first procedure adopted in clinical practice and is regarded as safe and effective, and the preferred initial treatment option irrespective of etiology [2, 7–9]. However, the procedure sometimes required frequent repetition due to a high risk of recurrence, and this severely influenced the patient's quality of life. Thus, a new therapeutic method is warranted to meet clinical demand.

In previous studies, oral administration and intralesional injection of corticosteroids have been used to soften scars and keloids with promising results, as it has pharmacological effects of inflammatory response inhibition and fibrotic tissue reduction [10, 11]. Some studies also investigated the efficacy of intralesional steroid injections for benign gastrointestinal strictures and proposed to augment the effect of ED [12–14]. Since the esophagus was a narrow tubular organ with a very high incidence and recurrence of stricture, local triamcinolone injection for esophageal strictures was supposed to reduce stricture recurrence by several studies [13, 14].

However, current studies about this issue were limited by small sample size or inconsistent data. We performed a meta-analysis including all prospective randomized controlled trials (RCTs) investigating the clinical efficacy and safety of intralesional triamcinolone injection (ITI) for benign esophageal strictures.

## 2. Methods

### 2.1. Inclusion Criteria

The following inclusion criteria were used to identify relevant studies: (1) Patients were individuals with benign esophageal strictures after surgery and/or corrosive injury. (2) Intervention was ITI in the treatment group, and comparison was saline injection (sham control) or no injection (blank control) in the control group. ED was performed conventionally mainly based on the demand of patients because of significant strictures (defined as failure of passing by an adult using a gastroscope of 8–9.8 mm diameter). (3) Outcome measures included stricture rate, ED sessions, dysphagia-free time, and treatment-related complications. Besides, clinical studies designed as RCTs were available without language limitation.

### 2.2. Search Strategy

Literature search was conducted in databases including MEDLINE (1966–Mar 2018), EMBASE (1978–Mar 2018), the Cochrane Library (1993–Mar 2018), and Web of Science (1985–Mar 2018). Search terms are as follows: (esophageal OR oesophagus OR esophagus) AND (stenoses OR stricture OR stenosis) AND (triamcinolone OR steroid OR corticosteroids injection). References of case reports, comparative studies, and reviews were also scanned to manually search relevant articles. Two reviewers independently reviewed the search results according to the inclusion criteria through screening the title and abstract. For potential studies, full-text papers were further evaluated independently for final inclusion. Disagreements between reviewers were resolved in consultation with a third reviewer (Zhang YC).

### 2.3. Data Extraction and Quality Assessment

Another two reviewers independently extracted the data including basic information, outcome measures, and methodological quality items. Any disagreements between the reviewers were resolved by discussion. Basic information included first author, publication year, sample size, average age of patients, intervention, comparison, dose of triamcinolone, diagnosis of patients, and follow-up periods. Outcomes included stricture rates, required dilation sessions, dysphagia-free time, and complications. Methodological quality items included randomization, allocation concealment, blinding, withdrawal and dropout, selective reporting result, and other biases. Quality assessment was performed independently by two reviewers according to the method and the tool of risk of bias recommended by the Cochrane Handbook [15].

### 2.4. Statistical Analysis

Review Manager (version 5.3, the Cochrane Collaboration, Copenhagen) was used to analyze data. For dichotomous outcomes, risk ratio (RR) with 95% confidence intervals (CI) was used. For continuous outcomes, standard mean difference (SMD) or mean difference (MD) were used. *P* < 0.05 was considered of statistical significance. The chi-square test was performed to assess the statistical heterogeneity across trials and *I*^2^ value to assess the extent of inconsistency. When *I*^2^ > 50%, the random-effect model was used. If *I*^2^≦50%, the fixed-effect model was applied. Publication bias was explored using an inverted funnel plot.

## 3. Results

### 3.1. Literature Search Result and Study Characteristics

**Figure 1:**
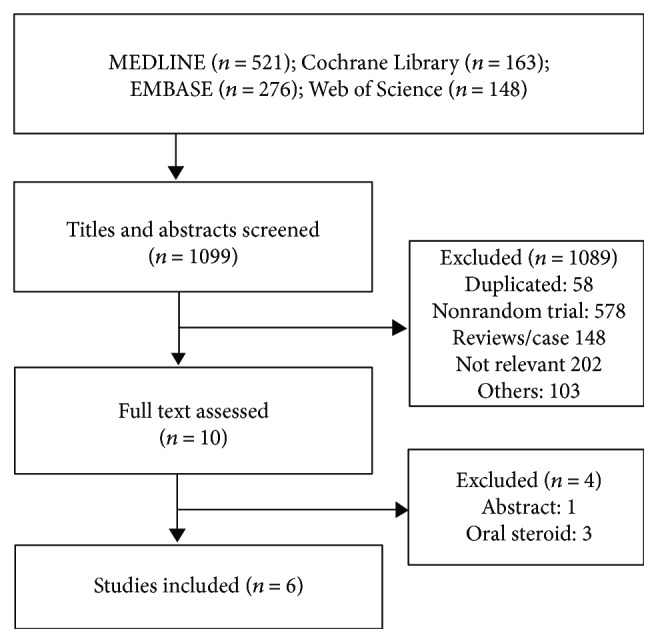
Flow chart of trial selection process.

**Figure 2:**
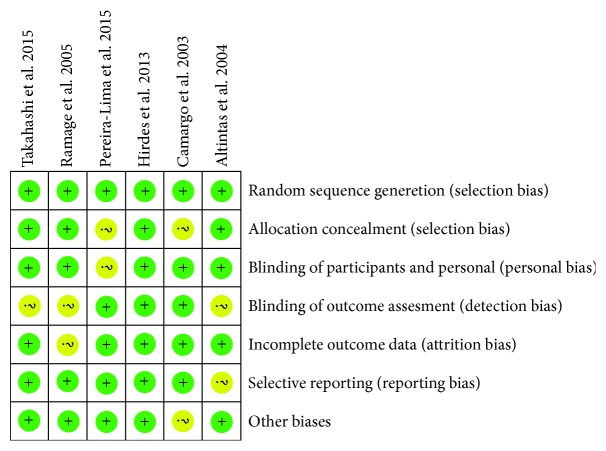
Summary of methodological quality of included studies.

We identified 1099 citations from online databases and obtained 10 full texts of articles based on the titles, abstracts, and full-text evaluation. Finally, six studies enrolling 176 patients were included for quantitative analysis [16–21], as shown in Figure 1. Basic information of the included RCTs was listed in Table 1. The sample size ranged from 14 to 60 patients. Two trials adopted a sham control with saline injection [19, 21], and four trials adopted no injection. Five trials adopted bougie dilation, and only one trial adopted balloon dilation [18]. In the study of Ramage et al. [18], the patients received dilation 1-2 times in the past 18 months, which was reported comparable between the groups. The doses of intralesionally injected triamcinolone ranged at 20 mg, 32 mg, and 40 mg per patient, and one trial injected 5 mg of triamcinolone every 10 mm point around the stricture. The diagnosis included surgical injury in three trials, peptic and corrosive injury in two trials, and both surgical and corrosive injury in one trial. Quality assessment was shown in Figure 2, and the overall quality was moderate to high.

### 3.2. Stricture Rate

Five studies reported the stricture rate after steroid injection during follow-up [16–20]. Significant stricture was defined as failure of passing by an adult using a gastroscope of 8–9.8 mm diameter and the demand of a repeated dilation. Meta-analysis in a fixed-effect model showed that ITI significantly reduced the incidence of stricture compared with control, and stricture rates were 50% and 78% in the groups.

**Figure 3:**
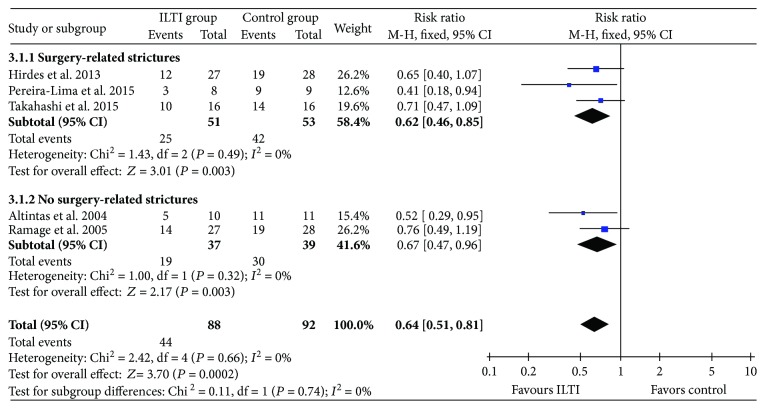
Forest plot of stricture rate between ITI and control.

Subgroup analysis according to different stricture etiologies showed that the risks of surgery-related strictures and non-surgery-related strictures were both reduced after ITI therapy, as shown in Figure 3.

### 3.3. Required ED Sessions

Four studies reported the number of required ED sessions during follow-up [16, 17, 19, 20]. Statistical heterogeneity was mild (*I*^2^ = 11%). Meta-analysis results showed that ITI significantly reduced the required ED sessions compared with the control.

**Figure 4:**
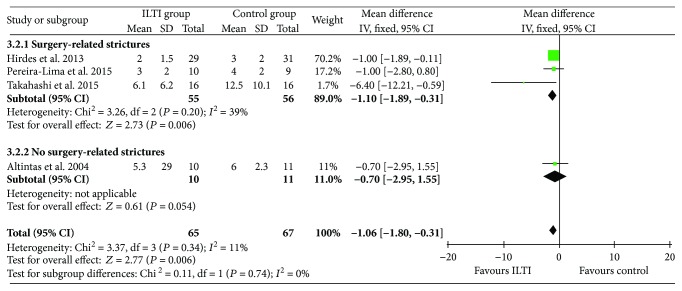
Forest plot of EBD sessions during follow-up between ITI and control.

Also, subgrouping according to different stricture etiologies showed that the number of required ED sessions was reduced after ITI therapy in the subgroup of surgery-related strictures. However, there was only one study including 21 patients in the subgroup of non-surgery-related strictures, and no significant difference was found, as shown in Figure 4.

### 3.4. Dysphagia-Free Time

Four studies reported the data of dysphagia-free time [16, 17, 19, 20]. There was a large heterogeneity across the trials (*I*^2^ = 88%), thus the random-effect model was used. No significant difference in dysphagia-free time was found between the groups.

**Figure 5:**
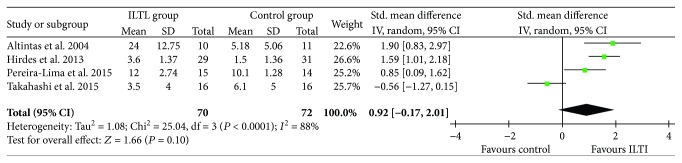
Forest plot of dysphagia-free time between ITI and control.

After excluding the study causing the large heterogeneity [16], the *I*^2^ value was reduced to 38% and fixed-effect model meta-analysis results of the remaining three studies showed that ITI significantly reduced the duration of dysphagia-free time compared with the control (Figure 5).

### 3.5. Complications

Injection-related complications were reported in two trials with a total of eight patients, and the others stated no related complications. Among them, two had perforations, one experienced bleeding, one had mucosal tearing, and four suffered from local infection. The adverse effects were similar between the patients treated with steroids and those without steroids. There was no statistically significant difference in the incidence of complications.

### 3.6. Publication Bias

**Figure 6:**
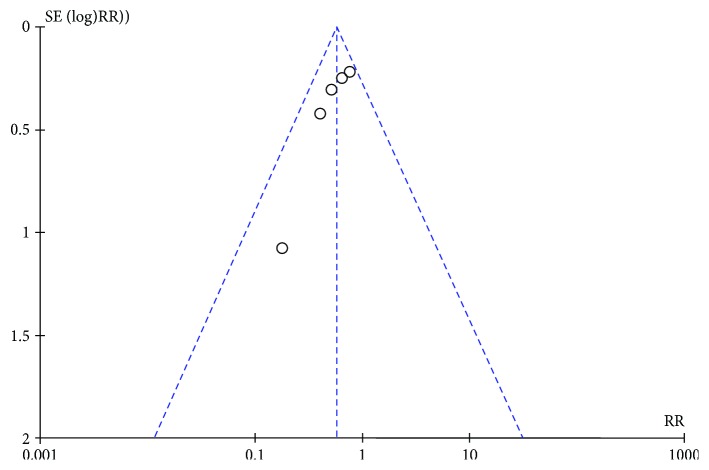
Inverted funnel plot of stricture rate.

Due to the limited number of included studies, a publication bias test through an inverted funnel plot was only adopted for the outcome of the stricture rate. The shape was to some extent symmetrical, indicating a lower risk of publication bias (Figure 6).

## 4. Discussion

A benign esophageal stricture was diagnosed by clinical, radiological, and endoscopical features and biopsies [16, 22]. Therapeutic options for a benign stricture included ED, temporary stent placement, intralesional steroid injection, and incisional therapy. Among these methods, ED is the cornerstone treatment [1]. Esophageal dilation was performed using either through-the-scope balloons or wire-guided bougies. A defined esophageal diameter to be targeted by dilation is different from patients with different severities, but the majority of patients have considerable symptomatic improvement when a diameter of 15–18 mm has been reached [16–19]. However, most of the intractable strictures are often unsuccessful with a high incidence of recurrence, which then require repeated dilations [23, 24]. This would seriously influence the quality of life and also increase the risk of complication in these patients. As estimated by the current study, the recurrence rate of stricture in the control group of benign esophageal stricture in a 6- to 12-month follow-up period was as high as 78%.

Various investigators investigated the role of corticosteroid injection into the stricture for the prevention of recurrent and complex strictures. Holder et al. were the first to report the use of intralesional steroid injection (ISI) into benign esophageal strictures of dogs and children, and the therapy was used only occasionally during the 1970s and 1980s [25, 26]. Over the last decade, increasing interests were presented in the use of the therapy for refractory benign esophageal strictures [12, 13, 16, 18].

Some other large-scale comparative studies reported their primary results and findings as follows. Kochhar et al. reported 71 patients with benign esophageal stricture receiving ISI; all categories of stricture that required ED sessions were significantly decreased, while the luminal diameter was increased. Interestingly, it also indicated that the location, number, and length of the stricture did not influence the efficacy of treatment [13]. Lee et al. reported a study of 31 patients, where all of them were diagnosed by endoscopy and treated with ED and steroid injection in each of the four quadrants at the narrowest region of the stricture [12]. The results showed that ISI led to symptomatic improvement and less frequent dilation. Furthermore, no complications were encountered. However, Camargo et al. did not find an improvement in dilation frequency or dysphagia in 14 patients with corrosive strictures allocated to steroid injections [21]. A study that included 21 patients with strictures of various etiologies receiving preventive ED found an increase in dysphagia-free period and periodic dilation index, while no difference in required dilations [16]. So, for the difference across the studies, study design, kinds of etiology, and dose of steroid would be all potential factors that influenced the clinical outcomes.

The present meta-analysis of the high quality of RCTs only investigated the benign esophageal stricture of surgical and corrosive injuries, and the results showed that additional ITI was more helpful than ED alone for the management of the strictures, as it reduced the stricture rate and number of required ED sessions during follow-up. Subgroup analysis for etiology of surgery- or non-surgery-related strictures showed that ITI therapy seemed to achieve even better results for surgery-related strictures in all outcomes. Thus, it is supposed that when endoscopic ITI was applied with a conventional intention of ED, the outcomes of stricture control as well as patients' quality of life would be significantly improved. Regretfully, the meta-analysis indicated that the dysphagia-free time might not be prolonged, as the dysphagia-free time was determined by the time when a patient felt dysphagia and came to visit the surgeons. Meanwhile, dysphagia is a very subjective complaint, and the tolerance levels across patients may be very different. Thus, such negative results would be caused by the situations and by the insufficient test power of the relatively small sample size.

Obviously, there were no life-threatening or serious complications that occurred in patients undergoing quadrant injection. Additionally, our study did not find a significant difference in reported injection-related complications such as perforation, bleeding, mucosal laceration, and local infection. However, it was reported that ISI may increase the risk of candidal esophagitis [18]. Due to the rare incidence of complications, as well as our relatively small sample size, the current conclusion should be considered carefully, and high-risk patients need to be evaluated thoroughly in clinical practice.

The limitation of this meta-analysis included the small sample size of participants, which might be insufficient to achieve very strong results in aspects of dysphagia-free time and complications. There are also some differences in the included studies: (1) Even though both surgical and corrosive strictures were benign, without clear resolution of the mechanism they still might have possible differences in pathogenesis and pathophysiology, and this gave rise to different prognoses and heterogeneities, although subgroup analysis was performed with no significant statistical difference. (2) The detailed ITI procedure was not completely the same, and this might also influence the outcomes, although the interventions in each trial were comparable. (3) Both bougie dilation and balloon dilation were used to conduct the dilation, which may also partly influence the treatment efficacy, which could be difficult to avoid in the follow-up periods after more than six months.

## 5. Conclusions

Additional ITI therapy was supposed to be effective and safe for the management of benign esophageal strictures as it reduced the stricture rate and required ED sessions. However, the relatively small sample size of participants was included especially in the evaluation of safety, and larger-scale RCTs are still needed to support the findings.

## Figures and Tables

**Table 1:** Characteristic of included randomized controlled trials.

Study	Country	Case (T/C, *n*)	Age (T/C, y)^∗^	Intervention (T/C)		Dose	Diagnosis	Follow-up (months)
				T	C			
Takahashi et al. 2015 [16]	Brazil	7/7	39 (23–64)/46 (22–65)	ED + ITI	ED + saline injection	40 mg	Corrosive stenosis	12
Altintas et al. 2004 [17]	Turkey	10/11	49 (24–69)/45 (17–76)	ED + ITI	ED	32 mg	Corrosive, surgical, postradiotherapy	>6
Ramage et al. 2005 [18]	USA	15/15	66/67	ED + ITI	ED	20 mg	Corrosive esophageal stricture	>12
Hirdes et al. 2013 [19]	Netherlands	29/31	64 ± 9/62 ± 8	ED + ITI	ED + saline injection	20 mg	Anastomotic stricture	6
Pereira-Lima et al. 2015 [20]	Brazil	10/9	56 ± 8/52 ± 15	ED + ITI	ED	40 mg	Anastomotic stricture	6
Camargo et al. 2003 [21]	Japan	16/16	70 ± 10/71 ± 7	ED + ITI	ED	>30 mg	Endoscopic surgery stricture	>16

^∗^Data were presented as mean ± standard deviation or median (range); T, treatment group; C, control group. ED, endoscopic dilation. ITI, intralesional triamcinolone injection.

## Abbreviations

ITI: Intralesional triamcinolone injection
ISI: Intralesional steroid injection
ED: Endoscopic dilation
RCTs: Randomized control trials
SMD: Standard mean difference
MD: Mean difference.
